# Autologous chondrocyte implantation for rheumatoid arthritis of the knee: a case report

**DOI:** 10.1186/1752-1947-3-6619

**Published:** 2009-04-03

**Authors:** Seok-Jung Kim, Cheong-Ho Chang, Dong-Sam Suh, Hyun-Kwon Ha, Kyung-Hwan Suhl

**Affiliations:** 1 Department of Orthopedic Surgery, College of Medicine, The Catholic University of Korea, Seoul, Korea; 2 RMS, SewonCellontech, Seoul, Korea; 3 Department of Radiology and Research Institute of Radiology, Asan Medical Center, Seoul, Korea

## Abstract

**Introduction:**

Although pharmacologic treatment remains the mainstay for treating rheumatoid arthritis, there is an increasing need for a method that biologically regenerates arthritic knee lesions as patient longevity continually increases.

**Case presentation:**

We treated rheumatoid arthritis of the right knee in a 35-year-old female Korean patient using autologous chondrocyte implantation. Twelve months after surgery, the patient could walk without pain.

**Conclusion:**

Autologous chondrocyte implantation appears to be effective for treating rheumatoid arthritis of the knee.

## Introduction

Rheumatoid arthritis (RA) is an autoimmune disease that causes chronic inflammation of the joints. To date, pharmacologic treatment remains the primary form of treatment, however, if pain and limitation of joint function become severe and debilitating, surgical treatment should be considered [1]. Over the last few decades, artificial joint replacement techniques have developed very rapidly and many arthritic conditions have thus been successfully treated [2]. However, as total joint arthroplasty is not permanent, in some cases, revisional surgery is inevitable, especially for young patients [3]. Therefore, there is an ever-increasing need for a method that biologically regenerates the arthritic lesion of the knee as patient longevity continually increases. This report presents the case of a 35-year-old woman with a painful, arthritic knee and her treatment using autologous chondrocyte implantation (ACI).

## Case presentation

**Figure 1:**
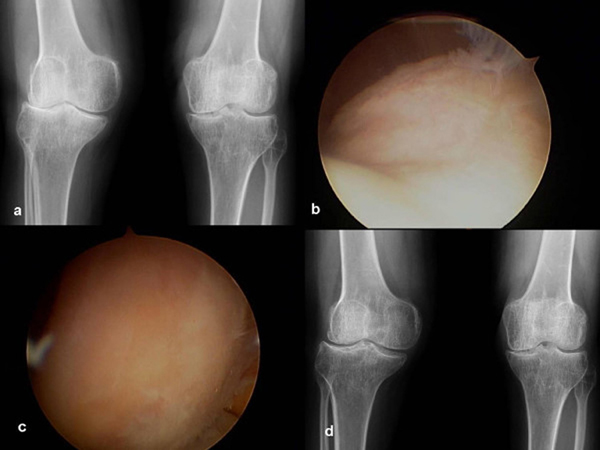
****(a)** Pre-operatively, both knee standing anteroposterior radiographs show lateral joint space narrowing and a 13° valgus deformity**. Arthroscopic findings of the **(b)** trochlea and **(c)** lateral condyle show severe arthritic change. **(d)** Both knee standing anteroposterior radiographs obtained 12 months after the autologous chondrocyte implantation surgery show widening of the lateral joint space and improvement in the valgus deformity to 7°.

A 35-year-old Korean woman with RA was admitted for right knee joint pain. Plain radiographs (Figure 1a) revealed progression of arthritis with lateral joint space narrowing when compared with radiographs obtained 4 years previously. The patient refused total joint replacement arthroplasty which our medical staff recommended and requested ACI. Knee arthroscopy was then performed to harvest 200g of autologous cartilage from the intercondylar notch (Figure 1b,c). The cartilage fragment was sent to a commercial cell culturing facility (SewonCellontech, Seoul) for processing.

Autologous chondrocyte implantation was performed 6 weeks after her initial surgery when 48 × 10^6^ chondrocytes had been cultured. Autologous chondrocytes were aseptically processed, suspended in sterile Dulbecco's Modified Eagles Medium (DMEM), and were supplied in single-use containers. Cell viability was greater than 97% before final packaging. Twelve months postoperatively, the lateral joint space of the knee had become wider (Figure 1d), and the patient could walk and otherwise function without pain.

**Figure 2:**
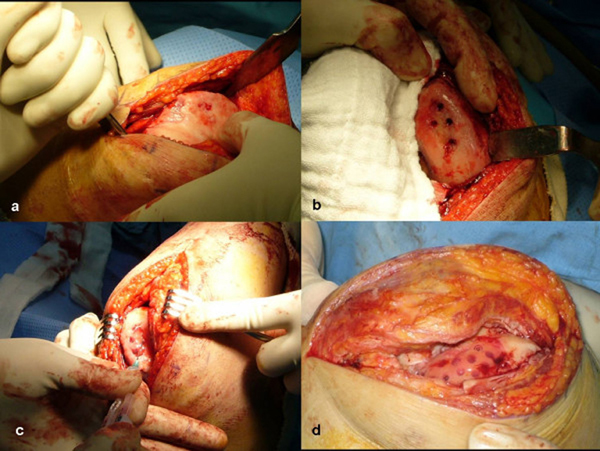
****Clinical photographs taken during the autologous chondrocyte implantation surgery:(a)** trochlea; **(b)** debridement and preparation of the femoral condyle; **(c)** injection of chondrocyte with fibrin into the prepared lesion; and **(d)** after injection of chondrocytes**.

With the patient under general anesthesia, a longitudinal, midline, skin incision was made, extending 5cm above the superior pole of the patella to the level of the tibia tubercle. The subcutaneous tissue was divided in the line of the skin incision. A medial skin flap was developed in order to expose the quadriceps tendon, medial border of the patella, and the medial border of the patellar tendon. A medial, parapatellar, capsular incision was made, and the patella was dislocated laterally to expose the arthritic chondral lesions (Figure 2a). The overhanging osteophytes were resected. Deformed and degenerated articular cartilage tissue was resected and debrided to the margin of the femoral condyles and patella. Multiple holes of 2mm depth and 2.5mm diameter were made at 1 to 2cm intervals using a 2.5mm drill bit, so that the holes of the defect would receive the holding force of the graft (Figure 2b). After release of the tourniquet, bone bleeding control was achieved using bone wax and compression force was applied to the holes using epinephrine-soaked gauze packing. For the injection procedure, two, 1-ml syringes and a Y-shaped mixing catheter were used. In one syringe, 1ml of fibrinogen (Tisseel, Baxter Inc., Korea) was filled with medium, and the other syringe was filled with 0.9ml of cell suspension and 0.1ml thrombin (50 IU). Cultured autologous chondrocytes mixed with fibrin (1:1) were then slowly injected into the defect area (Figure 2c,d).

In order not to overflow the margin, the dependent position of the defect site was maintained for 5 minutes. Flexion and extension motion of the knee was performed three to five times in order to check for any graft failure. The wound was then closed layer by layer.

The patient remained non-weight-bearing for 6 weeks postoperatively, then began bearing weight of approximately 10 to 15kg from the seventh post-surgical week, and gradually progressed to full weight-bearing at 12 weeks post-surgery. A range of motion exercises from 0° to 40° was started on the day following surgery using continuous passive motion (CPM) for 4 to 6 hours daily. After 1 week, the angle was increased by 5° per day. During this period, the quadriceps strengthening exercise and stretching of the hamstring and calf were continued.

## Discussion

RA is a systemic disease primarily affecting the synovium. When pharmacologic agents fail to control the inflammatory process of the synovium, symptomatic joint destruction begins. In addition, the surgical results have not been satisfactory for advanced disease which creates bony destruction [4].

Total joint replacement arthroplasty is the standard treatment for severe arthritis and deformity of the knee [5], but there is currently no successful treatment before the severe stage of RA. Many doctors simply prescribe medication and recommend that patients with knee pain wait until their symptoms increase to the point where total joint replacement arthroplasty becomes necessary.

The current indications for ACI do not include arthritis [6,7], as the present technique using liquid type chondrocytes cannot cover an arthritic lesion. In addition, there are many difficulties using the periosteum [8] to cover the total condyle so that it is watertight in the arthritic knee, and there is a high risk for breaking down of the treated lesion and progression to arthritis.

If we use a carrier, these problems can be overcome. Fibrin sealants are biological adhesives that mimic the final step of the coagulation cascade. They are used to reduce blood loss and postoperative bleeding [9]. In this patient, the fibrin can maintain the shape of the articulation approximately 5 minutes after injection, thus causing the cells to stay in the injected sites. Even if there is a defect along the chondral margin, fibrin helps to maintain the shape of the graft according to the articulation [10]. The multiple holes perform important functions by increasing the adhesive force of the graft to the defect during knee motion. In general, 2mm-deep holes in a sclerotic joint do not cause bleeding, however, in order to prevent both the formation of fibrocartilaginous tissue [11] and detachment of the injected cell and fibrin mixture, bleeding control is very important. The mild valgus deformity of this patient's knee was not corrected intra-operatively in order to minimize the potential for postoperative morbidity, and, fortunately, the deformity was naturally corrected as the lateral joint space became wider (Figure 1d) Our patient's symptom improvement was presumably due to coverage of the arthritic joint as well as new cartilage formation.

## Conclusion

Although, to date, our experience is limited to one patient, autologous chondrocyte implantation appears to be effective for treating rheumatoid arthritis of the knee.

## Consent

Written informed consent was obtained from the patient for publication of this case report and any accompanying images. A copy of the written consent is available for review by the Editor-in-Chief of this journal.

## Competing interests

The authors declare that they have no competing interests.

## Authors' contributions

SK was involved in patient care and drafting the manuscript as the main author. CC and HH were involved in defining the study concept and in the radiologic evaluation. DS was involved in the cell preparation and surgical technique protocol setup. KS was involved in the data analysis and literature review.
